# The Mitochondrial Fission Regulator DRP1 Controls Post-Transcriptional Regulation of TNF-α

**DOI:** 10.3389/fcimb.2020.593805

**Published:** 2021-01-14

**Authors:** Fushan Gao, Mack B. Reynolds, Karla D. Passalacqua, Jonathan Z. Sexton, Basel H. Abuaita, Mary X. D. O’Riordan

**Affiliations:** ^1^ Department of Microbiology and Immunology, University of Michigan Medical School, Ann Arbor, MI, United States; ^2^ Department of Orthopedics, The Second Xiangya Hospital, Central South University, Changsha, China; ^3^ Department of Internal Medicine, Gastroenterology, University of Michigan Medical School, Ann Arbor, MI, United States; ^4^ U-M Center for Drug Repurposing, University of Michigan, Ann Arbor, MI, United States; ^5^ Michigan Institute for Clinical and Health Research, University of Michigan, Ann Arbor, MI, United States; ^6^ Department of Medicinal Chemistry, College of Pharmacy, University of Michigan, Ann Arbor, MI, United States

**Keywords:** mitochondria, inflammation, macrophage, cellular stress, cytokine

## Abstract

The mitochondrial network plays a critical role in the regulation of innate immune signaling and subsequent production of proinflammatory cytokines such as IFN-β and IL-1β. Dynamin-related protein 1 (DRP1) promotes mitochondrial fission and quality control to maintain cellular homeostasis during infection. However, mechanisms by which DRP1 and mitochondrial dynamics control innate immune signaling and the proinflammatory response are incompletely understood. Here we show that macrophage DRP1 is a positive regulator of TNF-α production during sterile inflammation or bacterial infection. Silencing macrophage DRP1 decreased mitochondrial fragmentation and TNF-α production upon stimulation with lipopolysaccharide (LPS) or methicillin-resistant *Staphylococcus aureus* (MRSA) infection. The defect in TNF-α induction could not be attributed to changes in gene expression. Instead, DRP1 was required for post-transcriptional control of TNF-α. In contrast, silencing DRP1 enhanced IL-6 and IL-1β production, indicating a distinct mechanism for DRP1-dependent TNF-α regulation. Our results highlight DRP1 as a key player in the macrophage pro-inflammatory response and point to its involvement in post-transcriptional control of TNF-α production.

## Introduction

Tumor necrosis factor-α (TNF-α) is a potent pro-inflammatory mediator produced by macrophages upon infection to enhance host defense. TNF-α production is tightly regulated at multiple levels to minimize pathological inflammation and consequent tissue damage (Anderson, 2000). Dysregulated TNF-α levels are commonly linked to inflammatory diseases including systemic lupus erythematosus, septic shock, asthma, type II diabetes, and rheumatoid arthritis (Aggarwal et al., 2006). TNF-α engagement of its receptors, TNF-R1 and TNF-R2, mediates cell recruitment to sites of infection, immune cell activation, and production of antimicrobial molecules, cytokines, and can result in programmed cell death (Klebanoff et al., 1986; Bekker et al., 2001; Wajant et al., 2003). TNF-α-deficient mice are highly susceptible to infectious agents (Marino et al., 1997; Bean et al., 1999) and TNF-α neutralization therapy increases the risk of opportunistic infections and reactivation of latent tuberculosis (Weisman, 2002), underscoring the prominent role of TNF-α in immunity against microbial infection.

Recognition of microbial molecules or pathogen-associated molecular patterns (PAMPs) by Toll-like receptors (TLRs) triggers the canonical NFκB pathway, a primary driver of pro-inflammatory gene expression, including the genes encoding TNF-α, IL-6, and pro-IL-1β (Falvo et al., 2010; Liu et al., 2017). TNF-α transcription can be negatively regulated by multiple effectors including IRAK-M and BCL-3, which inhibit distinct steps of the TLR cascade to shutdown TNF-α expression (Kobayashi et al., 2002; Wessells et al., 2004). Post-transcriptionally, mRNA stability and translational silencing also modulate production of TNF-α. The 3′ untranslated region of *Tnfα* mRNA contains cis adenine and uridine-rich elements (ARE) where trans-acting factors such as the zinc-finger protein tristetraprolin (TTP) and TIA-1 bind to promote its degradation and translational arrest respectively (Anderson, 2000). Loss of these cis- or trans-acting elements in animal models of disease leads to TNF-α overproduction and chronic inflammation (Taylor et al., 1996; Kontoyiannis et al., 1999), demonstrating the importance of these regulatory mechanisms.

Mitochondria-associated proteins and molecules, like reactive oxygen species (MitoROS), regulate many aspects of innate immune signaling, but the extent to which the mitochondrial network plays a role in TNF-α expression or secretion is not well established. Stimulation of macrophages with LPS profoundly alters mitochondrial metabolism, shifting towards glycolysis for energy production and enabling the accumulation of metabolites like succinate, which promotes pro-IL-1β expression in a HIF1-α dependent manner (Tannahill et al., 2013). Generation of mitoROS and the subsequent release of mitoDNA promotes NLRP3 inflammasome activation and increased IL-1β and IL-18 production (Nakahira et al., 2011; Shimada et al., 2012; Zhong et al., 2018). This inflammatory process is exacerbated by depletion of autophagy proteins LC3B and Beclin-1, implicating mitophagy as a critical tuning mechanism to control mitochondria-dependent innate immune signaling (Nakahira et al., 2011). Moreover, evidence suggests that NLRP3 can be physically recruited to the mitochondrial outer membrane through association with the mitochondrial lipid cardiolipin, enabling inflammasome activation (Iyer et al., 2013; Bronner et al., 2015; Cassel et al., 2016). During viral infection, mitochondrial antiviral signaling protein (MAVS) nucleates the formation of a signaling complex upon RIG-like Receptor (RLR) stimulation that is required for induction of Type I interferon (Castanier et al., 2010; Hou et al., 2011; Stoolman and Chandel, 2019). Thus, mitochondrially associated proteins, lipids and metabolites facilitate innate immune signaling and cytokine production at both transcriptional and post-transcriptional levels, but to date, there is little evidence for a requirement for mitochondrial regulation in TNF-α production in response to infection.

Dynamic regulation of the mitochondrial network through fission and fusion is integral to network function during homeostasis, stress and inflammation (Friedman and Nunnari, 2014; Meyer et al., 2018). DRP1, a GTPase recruited to mitochondria at points of ER contact, oligomerizes on the outer mitochondrial membrane in a ring-like structure that constricts to divide mitochondrial network filaments (Friedman et al., 2011). DRP1 is a prerequisite for some aspects of mitochondrial quality control, as stress-induced fission generates smaller mitochondrial network fragments that can be substrates for the initiation of mitophagy (Breitzig et al., 2018; Yao et al., 2020). Indeed, DRP1 controlled the magnitude of NLRP3 inflammasome activation in response to stimulation by LPS+ATP (Park et al., 2015). Here we investigate the contribution of DRP1 to the pro-inflammatory cytokine response to lipopolysaccharide (LPS) or infection by the human pathogen, methicillin-resistant *Staphylococcus aureus* (MRSA), and find that DRP1 plays an unanticipated role in post-transcriptional regulation of TNF-α.

## Materials and Methods

### Cell Immortalization and Culture Conditions

Murine bone marrow-derived macrophages (BMDMs) were immortalized as previously described (Hornung et al., 2008; De Nardo et al., 2018). Briefly, recombinant Cre-J2 virus harboring the *v-raf* and *v-myc* oncogenes were produced in 3T3 fibroblasts grown in Complete Dulbecco’s Modified Eagle Medium (DMEM) supplemented with 10% heat-inactivated fetal bovine serum (FBS) and 50 U/ml of Penicillin and 50 µg/ml of Streptomycin. Culture supernatants containing Cre-J2 virus were filtered and stored at −70°C. Mouse femurs and tibias were flushed and cells were transduced with Cre-J2 virus in macrophage differentiation media (50% DMEM, 2 mM L-Glutamine, 1 mM Sodium Pyruvate, 30% L929-conditioned medium, 20% FBS, 50 U/ml of Penicillin and 50 µg/ml of Streptomycin). Immortalized macrophages were grown for at least 1 month before use in experiments to ensure immortalization was successful. L-929 cells were cultured in Minimum Essential Eagle Medium (MEM) supplemented with 2 mM l-glutamine, 1 mM sodium pyruvate, 1 mM non-essential amino acid (NEAA), 10 mM HEPES, and 10% FBS. All experiments were performed in DMEM supplemented with 2 mM l-glutamine, 1 mM Sodium Pyruvate and 10% FBS. When indicated, macrophages were treated with the following inhibitors; 10 µM Z-VAD-FMK (R&D Systems) and 10, 20, or 50 µM CsA (Millipore Sigma). All cells were incubated at 37°C in 5% CO_2_. All animals used to derive bone marrow for macrophage culture were housed in specific pathogen free facilities at the University of Michigan Medical School Unit for Laboratory Animal Medicine (ULAM) and treated humanely in accordance with an IACUC-approved protocol.

### Generation of DRP1 Knockdown Cells

Lentivirus was generated in HEK293T packaging cells grown in DMEM with 10% FBS. Viral particles were produced by transfecting the cells with pLKO.1 plasmid encoding *Drp1*-specific shRNA and a non-target control (NT-Control) along with the packaging plasmids (pHCMV-G, and pHCMV-HIV-1) (Kulpa et al., 2013) using FUGENE-HD transfection reagent (Promega). The mouse *Drp1*-specific shRNA plasmid with the sense sequence of (GGCAATTGAGCTAGCTATA) and the non-target control shRNA plasmid were purchased from Sigma-Aldrich. Cell-free viral supernatants were collected, filtered and used to transduce immortalized macrophages. Transduced cells were selected with puromycin (3 µg/ml) and resistant cells were grown and used for the experiments.

### Bacterial Culture and Macrophage Infection

USA300 strain LAC, a community associated methicillin-resistant *Staphylococcus aureus* (MRSA), was stored at −70°C in LB medium containing 20% glycerol. MRSA were streaked out and cultured in tryptic soy agar (Becton Dickinson). Selected colonies were grown for 18 h in liquid tryptic soy broth at 37°C with shaking (240 rpm). MRSA were centrifuged, washed, and re-suspended in phosphate buffered saline (PBS). Bacteria count was estimated based on OD_600_ and confirmed by enumerating colony forming units (CFU). Macrophages were infected with MRSA at a multiplicity of infection of 20 (MOI 20) for 1 h. Infected macrophages were washed 3× with PBS and cultured in media containing gentamicin (50 µg/ml) or Lysostaphin (10 U/ml) to kill extracellular bacteria.

### Immunoblotting Analysis

Macrophages were seeded in 6-well tissue culture treated plates at a density of 1×10^6^/well. On the next day, cells were washed 3× with PBS and lysed on ice for 15 min with 0.1% NP40 lysis buffer containing protease inhibitor cocktail (Roche). For protein phosphorylation immunoblot experiments, lysis buffer was supplemented with PhosSTOP™ phosphatase inhibitor (Roche). Cell lysates were collected, centrifuged and lysate supernatants were heated at 95°C for 15 min in Laemmli sample buffer (Laemmli, 1970). Proteins were separated by SDS-PAGE on gradient gels (4–20%, Bio-Rad), transferred onto nitrocellulose membranes and blocked with PBS containing 0.05% Tween-20 and 1.0% dry milk or 5.0% BSA (blocking buffer). Membranes were incubated with primary antibodies in a blocking buffer overnight at 4°C. After washing with PBS, membranes were incubated with secondary LI-COR antibody for 1 h at room temperature and visualized using the Odyssey Infrared Imaging System (Li-Cor Biosciences). The following antibodies were used: rabbit anti-DRP1 antibody clone D8H5 (1:1000 dilution, Cell Signaling, Cat# 5391S), mouse anti-GAPDH antibody clone 6C5 (1:2000 dilution, Santa Cruz, Cat# SC-32233), rabbit anti-phospho-DRP1 Ser635 antibody clone D9A1 (1:1000 dilution, Cell Signaling, Cat# 4494S), rabbit anti-phospho-DRP1 Ser656 antibody clone D3A4 (1:1000 dilution, Cell Signaling, Cat# 6319S), rabbit anti-MFF antibody clone E5W4M (1:1000 dilution, Cell Signaling, Cat# 84580), rabbit anti-MFN2 antibody clone D1E9 (1:1000 dilution, Cell Signaling, Cat# 11952S), mouse anti-OPA1 (1:1000 dilution, BD Biosciences, Cat# 612606), rabbit anti-TOM20 antibody (1:1000 dilution, Proteintech, Cat# 11802-1-AP), IRDye 800CW Goat anti-Mouse IgG antibody (1:10,000 dilution, LI-COR, Cat# P/N 925-32210), and IRDye 680RD Goat anti-Rabbit IgG antibody (1:10,000 dilution, LI-COR, Cat# 925-68071).

### Immunofluorescence Staining and Confocal Microscopy

Macrophages were seeded onto #1.5 glass coverslips in 6-well plates at a density of 10^6^/well. On the next day, cells were left untreated or stimulated for 4 h with 200 ng/ml LPS derived from *Salmonella minnesota* R595 (InvivoGen). Macrophages were fixed with 3.7% paraformaldehyde at room temperature for 20 min and permeabilized for 30 min with the wash buffer (PBS containing 0.1% Triton X-100) and blocked with 10% Normal Goat Serum control (Invitrogen) in the wash buffer. Coverslips were incubated with primary and then secondary antibody for 45 min at room temperature in a staining buffer (PBS, 0.1% Triton X-100 and 10% Normal Goat Serum). Antibody used are as follows: Rabbit anti-TOM20 antibody (1:100 dilution, Proteintech, Cat# 11802-1-AP); Mouse anti-MT-ND1 (Complex I) antibody clone 18G12BC2 (1:100 dilution, Thermo Fisher, Cat# 43-8800); Alexa Fluor-488 Goat anti-Mouse antibody (1:200 dilution, Thermo Fisher, Cat# A-11001); Alexa Fluor-594 Goat anti-Rabbit antibody (1:200, Thermo Fisher, Cat# A-11012). Cells were counterstained with CellTracker Red CMTPX (Thermo Fisher, C34552) and DAPI (Thermo Fisher, D1306) according to manufacturing protocol. Coverglasses were washed and mounted onto microscope slides using Prolong Diamond Antifade Mountant (Thermo Fisher, P36970). Cells were imaged at 100× magnification using a Yokogawa CSU-X1 Spinning Disk confocal microscope (Nikon). Images were analyzed using the open source softwares CellProfiler (see below) and Fiji image processing package. Background was subtracted from representative images using Fiji ImageJ (30 pixel rolling ball radius).

### Mitochondrial Morphology Analysis

We developed an image analysis pipeline to quantify mitochondrial morphology using the open source software CellProfiler (Carpenter et al., 2006). This analysis pipeline and technical annotations are available upon request. Briefly, nuclei were identified based on two-class Otsu segmentation in the DAPI image. Similarly, cell area was determined based on Cell Tracker Red staining. Single cells were identified based on expansion of nuclear objects to the cell perimeter, where each cell contains a single nucleus. Mitochondrial objects were identified using the Complex I image and categorized as fragmented or non-fragmented based on object size (<1 µm^2^) and form factor (circularity >0.6) thresholds. Additionally, contiguous mitochondrial objects were skeletonized using the skeletonize module to assess the average length of mitochondria. The number and area of mitochondrial objects and the length of the skeletonized mitochondrial network were quantified and related to cells using the “relate objects” module to generate single-cell measurements. This process was repeated across four independent experiments (25–60 cells per condition, each experiment) and average values per experiment are reported.

### Plasma Membrane Integrity and Cell Death

A cell impermeable nucleic acid binding SYTOX fluorescent dye (Thermo Fisher) was used for measuring loss of plasma membrane integrity, which is indicative of cell death. Macrophages were seeded onto black, clear-bottom 96-well tissue culture plates at a density of 5×10^4^/well. On the next day, the media were exchanged with 100 µl of media without phenol red containing 500 nM SYTOX Green. Equal volumes of media with specific treatment DMSO, LPS (200 ng/ml), MRSA (MOI 20), CCCP (5 µM) or NP-40 (2%) were used. The green fluorescence intensity was measured at 8 h using a BioTek microplate reader.

### Cellular Reduction State and Viability

Cell-permeable redox indicators Resazurin (Biotium, Cat# 30025-1) and WST-1 (Roche, Cat# 5015944001 were used according to manufacturer protocols to monitor cellular metabolic activity, which is indicative of cell viability. NT-Control and DRP1 KD macrophages were seeded onto 96-well plates for the Resazurin assay or 24-well plates for the WST-1 assay at a density of 5×10^4^ or 2.5×10^5^/well, respectively. On the next day, cells were left untreated (Mock) or stimulated with LPS (200 ng/ml) and cellular metabolic redox was monitored. Absorbance was measured at 570 nm and 600 nm for the reduced Resazurin (Resorufin) and at 440 and 600 nm for the reduced WST-1 (Formazan) using a BioTek microplate reader.

### ATP Rate Assays

The rate of ATP production from mitochondrial respiration and glycolysis was measured by Seahorse XF Real-Time ATP Rate Assay (Agilent, Cat# 103677-100) as previously described (Passalacqua et al., 2019). NT-Control and DRP1 KD macrophages were seeded onto Seahorse XF96 cell culture microplates at a density of 4 X 10^4^ per well. On the following day, media was replaced and the following experimental conditions were tested after 4 h treatment: untreated (Mock), LPS (500 ng/ml) or MRSA (MOI 20). Cells were washed with the assay culture media and placed in a non-CO_2_ incubator at 37°C for 45 min, and media were exchanged with fresh, warmed assay media and the rate of ATP production was monitored using the Seahorse XFe96 Analyzer. Data were analyzed by using the XF Real-Time ATP Rate Assay Report Generator.

### Cytokine Analysis

Macrophages were seeded onto 24-well plates at a density of 2.5 X 10^5^ per well. Cells were left untreated (Mock), stimulated with LPS (200 ng/ml), LPS+ATP or infected with MRSA (MOI 20). Culture supernatants were collected at 4, 8, or 24 h and the level of cytokines (TNF-α, IL-1β, and IL-6) were quantified by ELISA by the University of MIchigan Cancer Center Immunology Core.

### RNA Extraction and RT-qPCR

Macrophages were seeded onto 6-well tissue culture plates at a density of 1 X 10^6^ cells per well. Cells were left untreated (Mock), stimulated with LPS (200 ng/ml) or infected with MRSA (MOI 20) for 4 h. Cells were washed 3× with PBS containing calcium chloride and magnesium chloride (PBS+/+) and RNA was extracted using Zymo Research Corporation Direct-zol RNA MiniPrep Plus kit. RNA was quantified by nanodrop and normalized across all conditions. cDNA synthesis was performed using random hexamers (Thermo Fisher, Cat# N8080127) and murine leukemia virus reverse transcriptase (RT, Thermo Fisher, Cat# 28025021). Quantitative PCR was performed using PowerUp SYBR Green Master Mix (Thermo Fisher, Cat# A25741) with the following primers: *Tnfα* forward primer sequence 5′CTTCTGTCTACTGAACTTCGG3′, *Tnfα* reverse primer sequence 5′-CAGGCTTGTCACTCGAATTTTG-3′, *Il1β* forward primer sequence 5′-GCCCATCCTCTGTGACTCAT-3′, *Il1β* reverse primer sequence 5′-AGGCCACAGGTATTTTGTCG-3′, *Actb* forward primer sequence 5′-ATGGTGGGAATGGGTCAGAAGGAC-3′, and *Actb* reverse primer sequence 5′-CTCTTTGATGTCACGCAGGATTTC-3′. qPCR conditions were 95°C for 10 min, and 40 amplification cycles of 95°C for 10 s, 53°C for 45 s, and 72°C for 1 min.

### Intracellular TNF-α Staining and Flow Cytometry

Macrophages were seeded onto 6-well tissue culture plates at a density of 1 X 10^6^ cells per well. Macrophages were left untreated (Mock), stimulated with LPS (200 ng/ml) or infected with MRSA (MOI 20) in the presence of 2 µM monensin (Biolegend, Cat# 420701) to block protein transport. Cells were washed, collected in ice cold PBS and fixed with 3.7% paraformaldehyde at room temperature for 15 min. Cells were blocked and permeabilized with 1× intracellular staining perm wash buffer for 30 min (Biolegend, Cat# 421002). Cells were stained with APC anti-Mouse TNF-α Antibody (1:100 dilution, Biolegend, Cat# 506308) in an intracellular staining perm wash buffer for 30 min in the dark at room temperature. Cells were washed 2× with 2 ml of intracellular staining perm/wash buffer and measured by flow cytometry (FACSCanto, BD Biosciences). Data was analyzed by FlowJo software. The mean fluorescence intensity for each condition was determined as the geometric mean.

### Statistical Analysis

Data were analyzed using Graphpad Prism 8.0. Unless otherwise stated, graphs are presented as a mean of n ≥ 3 independent experiments with standard deviation (SD) error bars. Differences between paired groups were tested using Two-way ANOVA and followed up by Sidak’s multiple comparisons test. *P* values < 0.05 were considered significant and designated by: *P < 0.05, **P < 0.01, ***P < 0.001 and ****P < 0.0001. All statistically significant comparisons are marked.

## Results

### Toll-Like Receptor Signaling Augments DRP1-Dependent Mitochondrial Fragmentation

**Figure 1:**
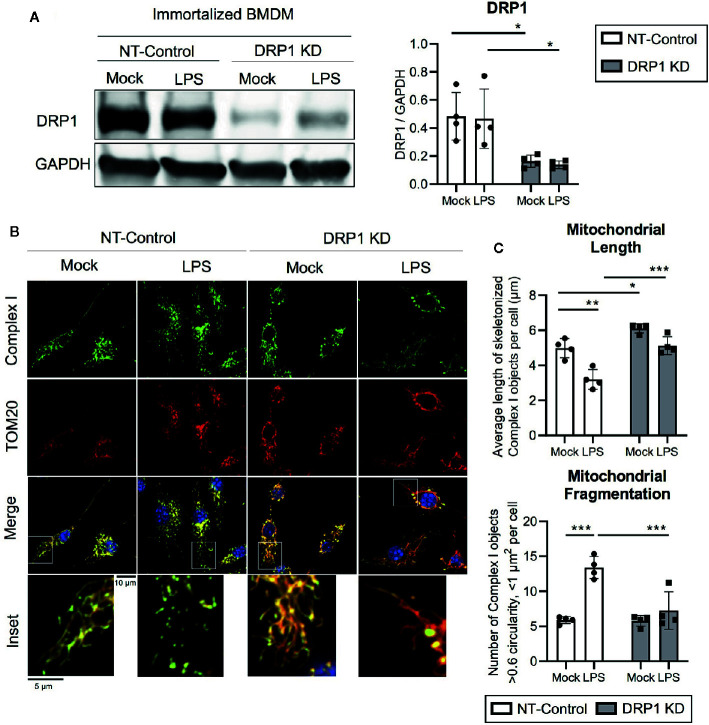
DPR1 is required for LPS-induced mitochondrial fragmentation. **(A)** Representative immunoblot analysis and quantification of total DRP1 protein in NT-Control and DRP1 KD immortalized Bone marrow- derived macrophage cell lines which were left untreated (Mock) or stimulated for 4h with 200 ng/ml LPS. **(B)** Representative confocal immunofluorescence images of NT-control and DRP1 KD macrophages stained for TOM20 (red), Complex I (green) and DAPI (blue) after 4h LPS stimulation. **(C)** Quantification of mitochondrial length and fragmentation was performed based on Complex I images of Mock and LPS-stimulated macrophages using CellProfiler image analysis software. Graphs are presented as mean of n=4 independent experiments with standard deviation (SD) error bars. *P* value was calculated using two-way ANOVA with Sidak’s post-test for multiple comparisons. *P < 0.05; **P < 0.01; ***P < 0.001.

To evaluate the role of mitochondrial fission regulator DRP1 in the macrophage response to inflammatory stimuli, we generated stable DRP1 knockdown (DRP1 KD) and non-target control (NT-Control) cell lines by expressing shRNA specific for *Drp1* or a non-target sequence in immortalized mouse bone marrow-derived macrophages (iBMDM). DRP1 protein levels were evaluated by immunoblot and were depleted in DRP1 KD macrophages compared to NT-Control macrophages (**Figure 1A**). Of note, DRP1 was lowered at the protein level in primary differentiation macrophages compared to immortalized macrophages (**Figure S1A**), which is also observed in other senescent cells relative to dividing cells (Mai et al., 2010). To test whether immune signaling induces changes in mitochondrial dynamics, we stimulated NT-Control and DRP1 KD macrophages with the Toll-like Receptor (TLR) 4 agonist lipopolysaccharide (LPS) and analyzed mitochondrial morphology by immunofluorescence confocal microscopy. The mitochondrial network was visualized by staining with antibodies specific for the mitochondrial outer membrane (OM) protein TOM20 and mitochondrial inner membrane (IM) Respiratory Complex I (Complex I) (**Figure 1B**). Silencing macrophage DRP1 resulted in elongated, tubular mitochondrial network morphology, as previously observed (Park et al., 2015). In contrast, mitochondrial morphology of NT-Control macrophages presented along a spectrum of fragmented (spherical and separated mitochondria) to networked (elongated and fused mitochondria). In agreement with prior reports, mitochondrial morphology appeared to be more fragmented upon LPS stimulation in NT-Control macrophages compared to unstimulated conditions (Gao et al., 2017). However, the level of mitochondrial fragmentation remained unchanged in LPS-stimulated DRP1 KD macrophages. To quantify the effect of DRP1 depletion on LPS-induced mitochondrial fragmentation, we established an automated image analysis pipeline for measuring fragmented mitochondria using CellProfiler image analysis software (**Figure S2**). Fragmented mitochondria were defined based on the size and shape of mitochondrial Complex I objects, where objects smaller than 1 µm^2^ with circularity greater than 0.6 were considered fragmented mitochondria and larger, less circular objects were considered non-fragmented. Mitochondrial fragmentation was quantitatively increased in LPS-stimulated NT-Control macrophages, whereas LPS-stimulated DRP1 KD macrophages failed to significantly induce mitochondrial fragmentation (**Figures 1C**, **S2C, D**). The increase in mitochondrial fragmentation was not due to increased mitochondrial mass since TOM20 levels were unchanged in NT-Control macrophages during the 4 h of LPS stimulation (**Figure S1D**). We also performed skeletonization analysis of the mitochondrial network and measured the mean length of skeletonized mitochondria per cell to assess mitochondrial elongation and network complexity. We observed that LPS stimulation shortened the average mitochondrial length per cell, consistent with an increase in mitochondrial fission (**Figure 1C**). Further, DRP1 KD led to a global increase in mitochondrial length and disrupted LPS-induced shortening of mitochondria. In addition, DRP1 KD cells were reported to contain bulb-like mitochondrial structures (Mito-bulbs) where mitoDNA and IM Cytochrome C are concentrated, which we also observed for Complex I (**Figure 1B**) (Möpert et al., 2009; Ban-Ishihara et al., 2013). These bulb-like mitochondrial structures were more readily apparent when DRP1 KD macrophages were stimulated with LPS compared with untreated cells (Mock), suggesting that formation of mito-bulbs might be a consequence of failure to undergo DRP1-dependent fission in response to LPS-induced stress.

To investigate whether LPS-induced changes in mitochondrial morphology were due to DRP1 post-translational modification, we tested the effect of LPS stimulation on DRP1 phosphorylation at its activating site (Ser635) and inhibitory site (Ser656) during LPS stimulation. Similar to a recent report, we observed a trend in increased DRP1 Ser635 phosphorylation, yet no change in DRP1 Ser656 phosphorylation during 4 h LPS stimulation (**Figures S1B, C**) (Kapetanovic et al., 2020). Furthermore, we examined the effect of LPS stimulation on expression of the key DRP1 adaptor protein Mitochondrial Fission Factor (MFF) as well as the well-characterized fusion regulators Mitofusin-2 (MFN2) and OPA1 (**Figures S1B, C**). We did not detect any differences in abundance of these regulators of mitochondrial fission and fusion during LPS stimulation. However, we did observe a global increase in MFN2 expression in DRP1 KD macrophages, which may promote mitochondrial elongation and further limit LPS-induced mitochondrial fragmentation in DRP1 KD macrophages. Collectively, our data support a role for DRP1 in promoting mitochondrial fragmentation in LPS-stimulated macrophages.

### DRP1 Is Dispensable for Metabolic Switching and Cell Death During Toll-Like Receptor Signaling and Methicillin-Resistant *Staphylococcus aureus* Infection

**Figure 2:**
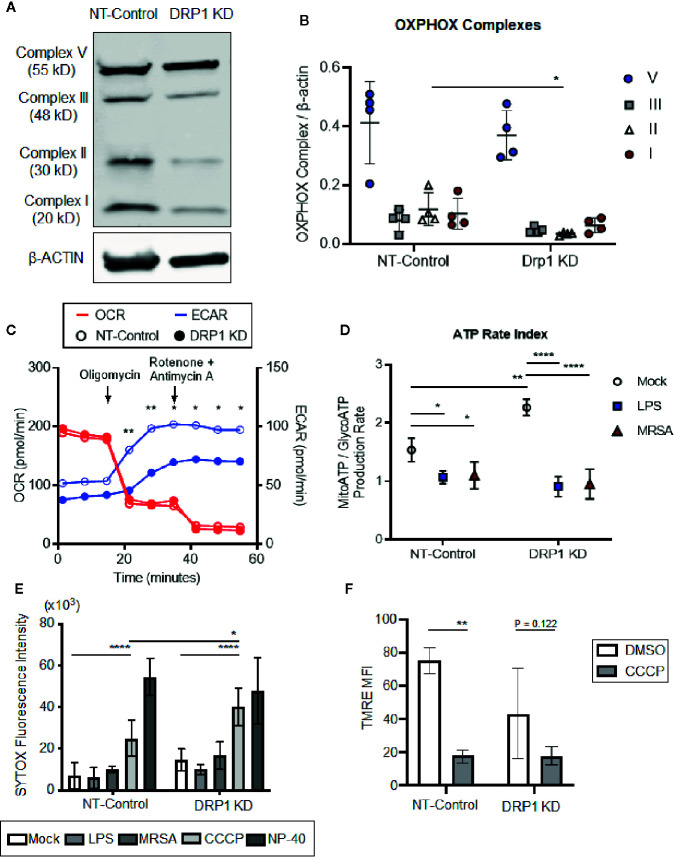
Silencing macrophage DRP1 does not influence metabolic reprogramming and cell death during immune stimulation. **(A)** Representative immunoblot of OXPHOX mitochondrial complexes protein expression from NT-Control and DRP1 KD macrophages. **(B)** Quantification of immunoblots from **(A)**. Graph symbols represent the ratio of OXPHOX Complex I-V expression levels relative to β-ACTIN of four independent experiments with mean ± SD. **(C)** Overlaid measurements of extracellular acidification and oxygen consumption rates (ECAR and OCR, respectively) using Agilent Seahorse ATP real-time rate assay in NT-Control and DRP1 KD macrophages. **(D)** Quantification of ATP rate index (a metabolic shift from mitochondrial respiration to glycolysis) in NT-Control and DRP1 KD macrophages when cells were left untreated (Mock), stimulated with LPS for 4h (LPS) or infected with MRSA at MOI of 20 for 4h (MRSA). ATP rate index is the ratio of the rate of mitochondrial ATP (MitoATP) production over glycolytic ATP (GlycoATP) production rate, which was quantified using the Agilent Seahorse ATP real-time rate assay. **(E)** SYTOX green fluorescence intensities of NT-Control and DRP1 KD macrophages were measured by plate reader after cells were exposed to DMSO, LPS (200 ng/ml), MRSA (MOI 20), CCCP (5 μM) or NP-40 (2%) for 8h. **(F)** Mitochondrial membrane potential was assessed by flow cytometry using TMRE dye. Mean fluorescence intensity (MFI) of NT-Control and DRP1 KD macrophages after 15min labeling with TMRE when cells were exposed for 4h to DMSO or CCCP (5 μM). MFI quantification was determined using FlowJo software, representing the geometric mean. Unless otherwise stated, graphs indicate mean ± SD of n ≥ 3 independent experiments. Two-way ANOVA followed by Sidak’s multiple comparisons test was performed to determine statistical significance. *P < 0.05; **P < 0.01; ****P < 0.001.

TLR signaling initiates a metabolic shift toward glycolysis and away from oxidative phosphorylation in macrophages, which enhances immunological responses, including production of proinflammatory cytokines (Kelly and O’Neill, 2015). Although mitochondrial morphology impacts metabolism (Yu et al., 2015), the role of DRP1 in metabolic reprogramming during immune stimulation in macrophages is poorly understood. To investigate whether depletion of DRP1 has consequences on LPS-stimulated metabolic reprogramming in macrophages, we first analyzed levels of mitochondrial oxidative phosphorylation (OXPHOS) complex subunits between DRP1 KD and NT-Control macrophages by immunoblot. We observed that depletion of DRP1 altered the stoichiometry of electron transport chain and ATP synthase complexes (**Figures 2A, B**). Alterations in the relative abundance of these respiratory complexes may influence their assembly into higher order supercomplexes to enhance respiration and ATP production by mitochondrial oxidative phosphorylation (Acín-Pérez et al., 2008). We therefore monitored the rate of mitochondrial and glycolytic adenosine triphosphate (ATP) production based on oxygen consumption rate (OCR) and extracellular acidification rate (ECAR) respectively in unstimulated, LPS-stimulated, or MRSA-infected macrophages using a Seahorse real time ATP rate assay (Romero et al., 2017). MRSA infection was included to represent a more physiological and dynamic interaction between host and pathogen. In unstimulated conditions and under steady state, DRP1 KD macrophages had a slightly lower ECAR and higher OCR when compared to NT-Control that resulted in a significantly higher calculated ratio of ATP produced by mitochondria to ATP produced by glycolysis (**Figures 2C, D**). Remarkably, when cells were treated with Oligomycin (Complex V inhibitor) and Rotenone (Complex I inhibitor) + Antimycin A (Complex III inhibitor) to inhibit mitochondrial ATP production, DRP1 KD cells produced substantially lower ECAR compared to NT-Control cells (**Figure 2C**), indicating that DRP1 is required for metabolic reprogramming in response to mitochondrial perturbations.

However, upon LPS stimulation, the ratio of ATP produced by mitochondria relative to glycolysis was decreased in both DRP1 KD and NT-Control macrophages, suggesting that DRP1 is not required for the LPS-induced metabolic shift toward glycolysis (**Figure 2D**). Similar results were observed in MRSA-infected macrophages, which shifted metabolic flux toward glycolysis independently of DRP1.

DRP1-mediated mitochondrial fission is a critical step in initiating cell death in response to stress stimuli such as staurosporine and ROS (Frank et al., 2001; Suen et al., 2008). Because macrophages stimulated with LPS or infected with MRSA induce mitoROS generation (West et al., 2011; Abuaita et al., 2018), we monitored susceptibility of DRP1 KD and NT-Control macrophages to cell death in response to immune stimulation or treatment with the mitochondrial oxidative phosphorylation uncoupler, carbonyl cyanide *m*-chlorophenyl hydrazine (CCCP). Cell death was assessed through uptake of the cell-impermeant nucleic acid fluorescent probe, SYTOX Green, which indicates loss of plasma membrane integrity (**Figure 2E**). SYTOX fluorescence intensity remained low upon LPS treatment or MRSA infection, comparable to untreated cells, indicating minimal cell death in DRP1 KD or NT-Control macrophages. In addition, we monitored cellular redox state using resazurin and WST-1 assays (O’brien et al., 2000; Berridge et al., 2005). DRP1 KD cells exhibited slightly lower levels of reduced resazurin and WST-1 compared to NT-Control cells, but stimulation with LPS did not lead to changes in cellular reduction status relative to unstimulated mock cells (**Figure S3**). However, we found that DRP1 blocked CCCP-induced cell death. Treatment with CCCP increased SYTOX fluorescence intensity in DRP1 KD relative to NT-Control macrophages even though CCCP treatment disrupted mitochondrial membrane potential similarly in both cell lines (**Figure 2F**). These results support a pro-survival role for DRP1 when mitochondrial membrane potential is compromised, likely through clearance of damaged mitochondria by mitophagy (Twig et al., 2008).

### DRP1 Restrains Macrophage IL-1β Production but Is Required for TNF-α Production

**Figure 3:**
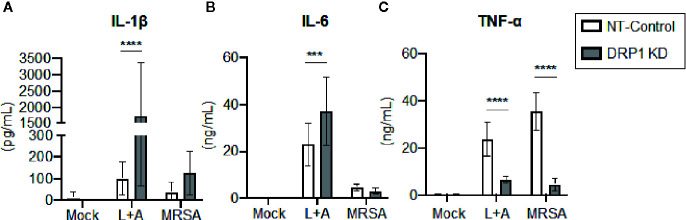
DRP1 blocks IL-1β and promotes TNF-α production during MRSA infection and sterile inflammation. Levels of IL-1β **(A)**, IL-6 **(B)** and TNF-α **(C)** in culture media of NT-Control and DRP1 KD macrophages were quantified by ELISA at 24h post stimulation. Macrophages was left untreated (Mock), stimulated with 200 ng/ml of LPS for 23h followed by 1 mM of ATP for 1h (L+A), or infected with MRSA (MOI 20). Graphs indicate the mean of n ≥ 4 independent experiments ± SD. Statistical analysis was performed by two-way ANOVA followed by Sidak’s multiple comparisons test. ***P < 001; ****P < 0.0001.

Recent evidence supports that DRP1 contributes to NLRP3 inflammasome activation in macrophages, since DRP1 KD in macrophages results in increased inflammasome activation and secretion of IL-1β (Park et al., 2015). The role of DRP1 in the regulation of other macrophage pro-inflammatory cytokines is poorly defined. To investigate the contribution of DRP1 to pro-inflammatory cytokine responses, we stimulated DRP1 KD and NT-Control macrophages with the classical inflammasome activation signals of LPS+ATP or MRSA infection. Following 24-h stimulation, we analyzed culture supernatants by ELISA to determine secretion of the pro-inflammatory cytokines IL-1β, IL-6, and TNF-α (**Figure 3**). We observed an increase in IL-1β secretion in DRP1 KD macrophages compared to NT-Control macrophages during inflammasome activation, comparable to previous reports (Park et al., 2015) (**Figure 3A**). To further investigate whether DRP1 impedes IL-1β production by decreasing expression *Il1β* transcriptionally, we monitored the levels of *Il1β* transcript in DRP1 KD and NT-Control macrophages in response to LPS stimulation by RT-qPCR (**Figure S4**). Similar levels of *Il1β* transcript were induced by both DRP1 KD and NT-Control macrophages in response to LPS stimulation or MRSA infection, suggesting that DRP1 inhibits IL-1β production independent of *Il1β* transcription. We also observed a slight increase in IL-6 secretion during inflammasome activation, but not MRSA infection by DRP1 KD macrophages, suggesting DRP1 may modulate IL-6 responses under some conditions (**Figure 3B**). Surprisingly, we observed a marked reduction in TNF-α production in DRP1 KD macrophages compared to NT-Control macrophages during both classical inflammasome activation and MRSA infection (**Figure 3C**). These findings suggest that DRP1 may regulate macrophage pro-inflammatory cytokine responses through multiple mechanisms, enhancing TNF-α and restraining IL-1β production.

### Silencing DRP1 Diminishes TNF-α Protein Levels

**Figure 4:**
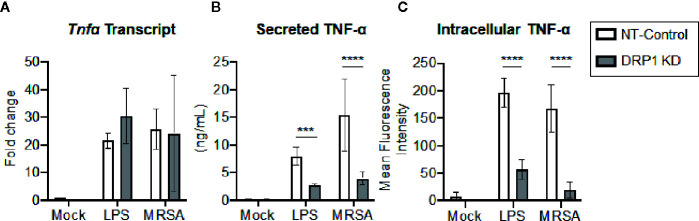
DRP1 regulates TNF-α production at post-transcriptional level. **(A)** Quantitative real-time PCR of *Tnfa* transcript levels of NT-Control and DRP1 KD macrophages after 4h stimulation with LPS or MRSA infection (MOI 20). Data are relative to NT-Control macrophage untreated (Mock) condition. **(B)** TNF-α levels in culture media of conditions from **(A)** were quantified by ELISA at 24h post-stimulation. **(C)** Total TNF-α levels were monitored by intracellular staining using flow cytometry after blocking protein transport with monensin. Macrophages were stimulated with LPS (200 ng/ml), infected with MRSA (MOI 20) or left untreated (Mock) in the presence of monensin 4h and stained intracellularly for TNF-α. Mean fluorescence intensity (MFI) of TNF-α was determined using FlowJo software, representing the geometric mean. Graphs show the mean ± SD from at least three independent experiments. *P* value was calculated using two-way ANOVA with Sidak’s post-test for multiple comparisons. ***P < 0.001; ****P < 0.0001.

Transcriptional and post-transcriptional events are known to regulate TNF-α production (Sariban et al., 1988; Anderson, 2000). We therefore evaluated whether DRP1 exerted an effect on TNF-α transcription, translation, and/or secretion. The cellular levels of *Tnfα* mRNA and production of mature TNF-α in culture supernatants were monitored simultaneously at 4 h post LPS-stimulation or MRSA infection (**Figures 4A, B**). The levels of *Tnfα* transcript were not changed by silencing DRP1, but TNF-α secretion in culture supernatants was decreased, suggesting that DRP1 regulates TNF-α production at the post-transcriptional level. Next, we assessed whether silencing DRP1 affected TNF-α protein levels by performing intracellular staining on macrophages treated with monensin to block protein secretion (**Figure 4C**). LPS treatment or MRSA infection of NT-Control macrophages induced accumulation of intracellular TNF-α protein in monensin-treated cells. In contrast, DRP1 KD cells failed to accumulate intracellular TNF-α protein, suggesting that DRP1 is required for TNF-α translation or stability.

### DRP1 Regulates TNF-α Levels Independently of Mitochondrial Outer Membrane Permeability

**Figure 5:**
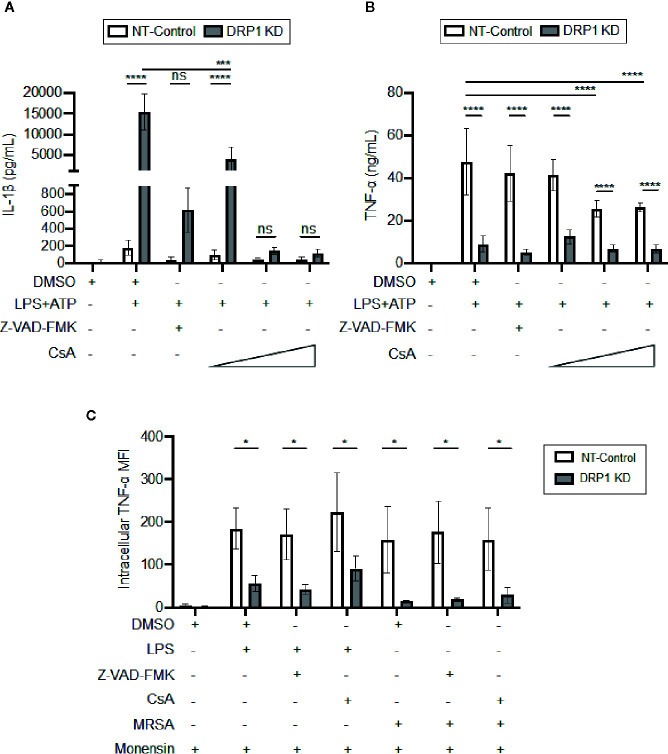
CsA blocks IL-1β production in DRP1 KD macrophages but inhibits TNF-α production by DRP1 independent mechanism. **(A)** Quantification of IL-1β **(A)** and TNF-α **(B)** levels in culture media of NT-Control and DRP1 KD macrophages after stimulation with LPS+ATP for 8h in the presence and absence of Z-VAD-FMK or various concentrations of CsA. **(C)** MFI of TNF-α intracellular staining of NT-Control and DRP1 KD macrophages was assessed by flow cytometry. Macrophages were treated with the indicated inhibitors and stimulated with LPS (200 ng/ml), infected with MRSA (MOI 20) or left untreated (Mock) for 4h. Data were analyzed with FlowJo software where MFI represents geometric mean. Graphs are presented as the mean of n ≥ 3 independent experiments ± SD. Two-way ANOVA with Sidak’s multiple comparisons post-test was used to determine statistical significance. *P <0.05; ***P <0.001; ****P <0.0001. ns: not significant.

DRP1 can induce release of Cytochrome C (CytC) into the cytosol to trigger caspase-mediated apoptosis in response to stress stimuli (Frank et al., 2001). Cyclosporine A (CsA), an inhibitor that prevents opening of the mitochondrial permeability transition pore (MPTP), blocks release of mitochondrial damage-associated molecular patterns (DAMPs) such as mitoDNA and cytC, which impact immune signaling and cytokine production (Broekemeier et al., 1989; Remick et al., 1989; Dawson et al., 1996; Kanno et al., 2002; Nakahira et al., 2015). Thus, we analyzed the effect of the MPTP blocker, CsA, or a broad-spectrum caspase inhibitor, Z-VAD-FMK, on cytokine production by DRP1 KD or NT-Control macrophages in response to LPS and ATP stimulation (**Figure 5A**). Treatment with Z-VAD-FMK or CsA caused reduction in the elevated level of IL-1β that was produced by DRP1 KD macrophages. Although the IL-1β baseline level produced by NT-Control was much lower than DRP1 KD macrophages, Z-VAD-FMK or CsA exposure also decreased IL-1β production by NT-Control macrophages to the level of unstimulated cells. In contrast, TNF-α produced by NT-Control macrophages was only reduced when macrophages were treated with higher concentrations of CsA, and Z-VAD-FMK treatment did not affect TNF-α levels at all (**Figure 5B**). To further investigate whether CsA blocks TNF-α translation similarly to DRP1 KD, we monitored the levels of cell-associated TNF-α by intracellular staining with protein transport inhibited by monensin (**Figure 5C**). CsA treatment did not decrease intracellular TNF-α in LPS-stimulated DRP1 KD or NT-control macrophages, revealing that CsA treatment might block TNF-α production by acting on a different stage of TNF-α regulation. Collectively, our data suggest that MPTP blockade restrains DRP1-dependent IL-1β responses but does not affect DRP1-dependent regulation of mature TNF-α.

## Discussion

Mitochondria are dynamic organelles that undergo fission and fusion processes to maintain cellular homeostasis. Previous studies have shown that DRP1 modulates production of cytokines such as IL-1β, IL-6, and IFN-β in response to immune stimulation and microbial infections (Castanier et al., 2010; Gao et al., 2017; Tiku et al., 2020). However, the mechanisms by which DRP1 regulates production of different pro-inflammatory cytokines are not well understood. In this study, we have shown that LPS stimulation triggers DRP1-dependent mitochondrial fragmentation, and that this mitochondrial fission regulator is required to maximally induce TNF-α and suppress IL-1β production. DRP1 expression was also required for MRSA-induced TNF-α production through post-transcriptional regulation, potentially implicating an effect of DRP1 on cytokine translation or stability. These data highlight the importance of DRP1 for mitochondrial fragmentation in response to innate immune stimulation and for post-transcriptional regulation of pro-inflammatory cytokine production.

Mitochondrial dynamics depend on the integration of complex cell-intrinsic and extrinsic cues. The functional consequences of reorganization of the mitochondrial network are context dependent, and the contributions of mitochondrial fission and fusion to the regulation of inflammatory responses remains controversial. Some studies report that pharmacological inhibition of the mitochondrial fission regulator DRP1 *in vivo* by the small molecule Mdivi-1 relieves inflammation and the production of pro-inflammatory cytokines in the context of inflammatory diseases (Fan et al., 2017; Li et al., 2019; Nair et al., 2019). Some *in vitro* studies support that macrophages genetically deficient in DRP1 secrete significantly more of the pro-inflammatory cytokine IL-1β due to increased activation of the NLRP3 inflammasome (Park et al., 2015), while others report that silencing DRP1 decreased LPS-induced inflammatory cytokine levels (Umezu et al., 2020). Therefore, contextual cues from specific cell lineages or genetic backgrounds may influence the role of DRP1 and mitochondrial fragmentation on inflammatory output. Indeed, primary mouse and human macrophages exhibit distinct metabolic shifts in response to stimulation by LPS (Vijayan et al., 2019), reinforcing the idea that mitochondrial control of inflammation is context-dependent. Our data show that genetic depletion of DRP1 results in diminished TNF-α production by macrophages in contrast to the heightened production of IL-1β. Since macrophages are major producers of TNF-α, these findings lend new genetic insight into the attenuation of inflammation observed *in vivo* upon pharmacological inhibition of DRP1 (Grivennikov et al., 2005). Notably, diminished IL-1β production is also observed *in vivo* during Mdivi-1 inhibition of DRP1 (Deng et al., 2020). While these differences may result from DRP1-independent, off-target effects of Mdivi-1 treatment on mitochondrial metabolism, it is also reasonable that loss of TNF-α production during Mdivi-1 treatment may mask the effect on IL-1β production *in vivo* (Bordt et al., 2017). Overall, these observations support that DRP1 is critical for the maintenance of pro-inflammatory cytokine responses during sterile inflammation.

The balance between mitochondrial fission and fusion can shift in response to infection to enhance macrophage effector function. Importantly, the outcome of alterations in the mitochondrial network is not conserved. Previous studies have observed that infection with bacterial pathogens is commonly associated with mitochondrial fragmentation, whereas viral infection often promotes mitochondrial fusion in macrophages (Tiku et al., 2020). Surprisingly, these polarized morphological phenotypes can converge to promote inflammation, albeit by different mechanisms. For example, some evidence supports that mitochondrial fission is important for IL-1β production during bacterial infection. In this model, mitochondrial DAMPs, such as mitoDNA or mitoROS, undergo a fission-dependent increase in abundance or cytosolic exposure to promote activation of the NLRP3 inflammasome (Park et al., 2015). At the same time, recognition of viral RNA by cytosolic RLRs can promote mitochondrial fusion, the activation of the MAVS, and MAVS-dependent NLRP3 inflammasome activation (Castanier et al., 2010; Park et al., 2013). In this way, both mitochondrial fission and fusion promote context-dependent macrophage effector function.

While some pathogen-derived virulence factors can reshape the mitochondrial network to promote infection, recent evidence suggests that signaling through pattern-recognition receptors, such as TLRs and RLRs, also plays an important role in defining mitochondrial dynamics (Castanier et al., 2010; Stavru et al., 2013; Gao et al., 2017; Rozzi et al., 2018). Consistent with previous reports, we have shown that TLR4 signaling via LPS stimulation enhances mitochondrial fragmentation (Gao et al., 2017; Nair et al., 2019; Kapetanovic et al., 2020). Furthermore, we have shown that knockdown of DRP1 disrupts LPS-induced mitochondrial fragmentation. However, additional studies are needed to determine the mechanism by which TLR4 signaling influences mitochondrial fragmentation. It is plausible that TLR4 signaling leads to rewiring of cellular metabolism to favor glycolysis, which can drive mitochondrial fragmentation due to concomitant accumulation of MitoROS (Willems et al., 2015; Mills et al., 2016; Lauterbach et al., 2019). Additionally, mitoROS may exacerbate NF-κB activation, thereby accelerating ROS production in a positive feedback loop leading to further mitochondrial fragmentation (Mariappan et al., 2010; Morgan and Liu, 2011; Wang et al., 2014). It has been shown that DRP1 activity can be regulated through activation of kinases ERK1/2 and NF-κB inducing kinase (NIK), which phosphorylate the activating site of DRP1, Ser616 (mouse Ser635) (Jung et al., 2016; Prieto et al., 2016). At the same time, Calcineurin and Protein phosphatase 2A can dephosphorylate the DRP1 inhibitory site, Ser656, to promote DRP1 function (Cribbs and Strack, 2007; Cereghetti et al., 2008; Merrill and Slupe, 2013). Our finding that DRP1 KD prevents LPS-induced mitochondrial fragmentation supports that DRP1 activity is critical for rapid reorganization of the mitochondrial network to generate a pool of fragmented mitochondria in response to inflammatory stimuli. Although we observed a trend in increased DRP1 Ser635 phosphorylation during 4 h LPS stimulation, it is not clear if this subtle change is sufficient to account for LPS-induced rearrangements in mitochondrial morphology. Still, it is possible that more robust phosphorylation of DRP1 occurs earlier during immune activation, as has been previously described for LPS stimulation (Kapetanovic et al., 2020). Finally, other post-translational modifications, including S-nitrosylation, SUMOylation, and ubiquitination, are known to regulate DRP1 function (Otera et al., 2013), yet the relevance of these modifications to LPS-induced mitochondrial fragmentation remain to be elucidated.

To our knowledge, a role for DRP1 in post-transcriptional control of TNF-α production has not been described. There are multiple known mechanisms by which TNF-α responses can be controlled following transcription. Specifically, post-transcriptional regulation of TNF-α production can occur at the level of mRNA nuclear export, translation, and protein stability (Anderson, 2000; Piecyk et al., 2000; Skinner et al., 2008; Lahat et al., 2008). As described above, ERK1 is a key regulator of DRP1 activity, yet it also critical for the control of *Tnfα* mRNA translation through activation of TAP and NXT1, RNA-binding proteins (RBPs) which facilitate nucleocytoplasmic transport (Skinner et al., 2008). Still, there is an incomplete link between ERK1 and the activity of these RBPs. It is plausible that ERK1 acts through DRP1 to control TNF-α production in this manner. In fact, mitochondria are known to engage in post-transcriptional regulation of many genes, particularly those involved in mitochondrial metabolism and biogenesis (Pearce et al., 2017). Further, another RBP TIA-1 acts by stalling translation and has been linked to both the post-transcriptional regulation of TNF-α production and the regulation of the MFF, which promotes DRP1 mitochondrial recruitment (Piecyk et al., 2000; Tak et al., 2017). Nevertheless, a role for TIA-1 in DRP1-dependent TNF-α production has not been identified. Finally, there is evidence that TNF-α can be degraded within macrophage lysosomes under certain stress conditions, such as hypoxia (Lahat et al., 2008). However, a mechanism by which mitochondria may engage in this process is not clear. Altogether, TNF-α is a potent inflammatory signal which is regulated at every level from transcription to secretion in macrophages. Perhaps not surprisingly, mitochondria, the hub for cellular metabolism and stress responses, may also regulate TNF-α production, perhaps through the process of DRP1-dependent mitochondrial fission. Mitochondrial constituents, such as mitochondrial DNA, have been implicated in chronic autoimmune diseases and may serve as the source of self-antigen in lupus (Kim et al., 2019), pointing to the concept of “friendly fire” as a principle in understanding endogenous disease drivers (Gomes-Leal, 2019). More broadly, regulators of mitochondrial stress and integrity, like DRP1 may represent under-appreciated targets for intervention in diseases with an inflammatory component, which include autoimmune syndromes as well as cardiovascular disease and neurodegenerative disorders (Meyer et al., 2018).

## Data Availability Statement

The raw data supporting the conclusions of this article will be made available by the authors, without undue reservation.

## Ethics Statement

The animal study was reviewed and approved by University of Michigan Institutional Animal Care & Use Committee.

## Author Contributions

FG, MR, KP, JS, BA, and MO designed, performed, and analyzed the experiments. FG, MR, JS, BA, and MO wrote the manuscript. All authors contributed to the article and approved the submitted version.

## Funding

This research was supported by the following awards from the National Institutes of Health: R21AI101777 (MO) and by R01DK120623 (JS). We gratefully acknowledge the Global Reach Program at University of Michigan Medical School for supporting FG. We also thank the University of Michigan Graduate Program in Immunology for supporting MR through the Research Training in Experimental Immunology training grant T32 AI007413. Research reported in this publication was supported by the National Cancer Institute of the National Institutes of Health under Award Number P30CA046592 by the use of the following Cancer Center Shared Resource(s): Immune Monitoring Core. The content is solely the responsibility of the authors and does not necessarily represent the official views of the National Institutes of Health.

## Conflict of Interest

The authors declare that the research was conducted in the absence of any commercial or financial relationships that could be construed as a potential conflict of interest.

## Supplementary Material

Supplementary Figure 1 Immunoblot analysis of mitochondrial fission and fusion machinery in macrophages during LPS stimulation. **(A)** Immunoblot comparing DRP1 protein content in primary differentiation BMDM vs. DRP1 KD and NT-Control immortalized BMDM +/- 4 h LPS stimulation (200 ng/ml). Lysates from equivalent numbers of cells (7.5×10^4^) were loaded to control for cell type-intrinsic differences in protein content. **(B)** DRP1 abundance was quantified relative to mitochondrial content (TOM20) based on immunoblots. **(C)** Analysis of mitochondrial fission and fusion regulators in 4 h LPS-stimulated macrophages by immunoblot. The abundance of DRP1 activating phosphorylation at Ser635, DRP1 inhibitory phosphorylation at Ser656, Mitochondrial fission factor (MFF), key mitochondrial fusion proteins, Mitofusion 2 (MFN2) and Optic atrophy 1 (OPA1), and mitochondrial OM marker TOM20 and matrix marker Pyruvate Dehydrogenase (PDH) were assessed. **(D)** Quantification of DRP1 Ser635 and Ser656 phosphorylation relative to total DRP1 (tDRP1) and TOM20, MFN2, OPA1, and MFF relative to GAPDH. * Graphs are presented as the mean of n ≥ 3 independent experiments ± SD. Two-way ANOVA with Sidak’s multiple comparisons post-test was used to determine statistical significance.*P* < 0.05; ***P* < 0.01; ****P* < 0.001.

Supplementary Figure 2 Quantification of mitochondrial morphology using CellProfiler. **(A)** CellProfiler workflow for mitochondrial fragmentation and skeletonization analysis of Complex I immunofluorescence images. **(B)** Mitochondrial and total cell objects were segmented based on Complex I and Cell Tracker Red CMTPX stains, respectively. Representative processed images of NT-Control and DRP1 KD macrophages showing fragmented mitochondria in red and non-fragmented mitochondrial objects in teal overlaid on Complex I images. Similarly, skeletonized mitochondrial networks (red) are shown overlaid on Complex I images. Cells were left untreated (Mock) or stimulated with LPS (200 ug/ml) for 4 h. **(C)** Quantification of mitochondrial fragmentation and length at the cell level. Fragmented mitochondria were classified based on mitochondrial object size and circularity. MitochondriaI objects with area less than 1 µm^2^ and a circularity greater than 0.6 were considered fragmented for the purpose of quantification. Cell-level measurements of the number of fragmented mitochondrial objects, percentage of the mitochondrial area classified as fragmented, and the mean length of mitochondrial skeletons are reported as violin plots with the mean designated by a red line. **(D)** Area of total and fragmented mitochondrial objects per cell are reported as the experimental mean. Similarly, the mean percentage of mitochondrial objects classified as fragmented per cell is reported for each experiment. Graphs are presented as the mean of >100 cells per condition across 4 independent experiments ± SD. Since cell-level data did not follow a normal distribution, *P* values were calculated using direct comparison with the nonparametric Mann-Whitney/Wilcoxon rank sum test. Experimental averages were analyzed with two-way ANOVA with Sidak’s post-test for multiple comparisons. ***P* < 0.01; ****P* < 0.001; *****P* < 0.0001.

Supplementary Figure 3 Silencing DRP1 reduces cellular redox. NT-Control and DRP1 KD macrophages were left untreated (Mock) or stimulated with LPS (200 ng/ml) and cellular redox was assessed by using Resazurin **(A)** and WST-1 **(B)** substrates. Graphs indicate mean ± SD of n ≥ 3 independent experiments. Two-way ANOVA with Sidak’s multiple comparisons post-test was performed to calculate *P* values. ***P* < 0.01; *****P* < 0.0001.

Supplementary Figure 4 DRP1 knockdown does not affect LPS or MRSA-induced *Il1β* transcription in macrophages. DRP1 KD and NT-Control macrophages were stimulated for 4 h +/- LPS (200 ng/ml) or MRSA (MOI 20) and then the levels of *Il1β* transcript were analyzed by RT-qPCR. Data were normalized against NT-Control macrophage untreated (Mock) condition. Graphs indicate mean ± SD of n ≥ 3 independent experiments. Two-way ANOVA with Sidak’s multiple comparisons post-test was performed to calculate *P* values. **P* < 0.05.
