# Supplementary material for: The Mitochondrial Fission Regulator DRP1 Controls Post-Transcriptional Regulation of TNF-α
Source: Front Cell Infect Microbiol. 2021 Jan 14;10:593805. doi: 10.3389/fcimb.2020.593805 (PMC7840702; doi:10.3389/fcimb.2020.593805)
Supplement: Supplementary Figure 1 — Immunoblot analysis of mitochondrial fission and fusion machinery in macrophages during LPS stimulation. (A) Immunoblot comparing DRP1 protein content in primary differentiation BMDM vs. DRP1 KD and NT-Control immortalized BMDM +/- 4 h LPS stimulation (200 ng/ml). Lysates from equivalent numbers of cells (7.5×10^4^) were loaded to control for cell type-intrinsic differences in protein content. (B) DRP1 abundance was quantified relative to mitochondrial content (TOM20) based on immunoblots. (C) Analysis of mitochondrial fission and fusion regulators in 4 h LPS-stimulated macrophages by immunoblot. The abundance of DRP1 activating phosphorylation at Ser635, DRP1 inhibitory phosphorylation at Ser656, Mitochondrial fission factor (MFF), key mitochondrial fusion proteins, Mitofusion 2 (MFN2) and Optic atrophy 1 (OPA1), and mitochondrial OM marker TOM20 and matrix marker Pyruvate Dehydrogenase (PDH) were assessed. (D) Quantification of DRP1 Ser635 and Ser656 phosphorylation relative to total DRP1 (tDRP1) and TOM20, MFN2, OPA1, and MFF relative to GAPDH. * Graphs are presented as the mean of n ≥ 3 independent experiments ± SD. Two-way ANOVA with Sidak’s multiple comparisons post-test was used to determine statistical significance.P < 0.05; **P < 0.01; ***P < 0.001. [file Image_1.pdf]

Fig. S1

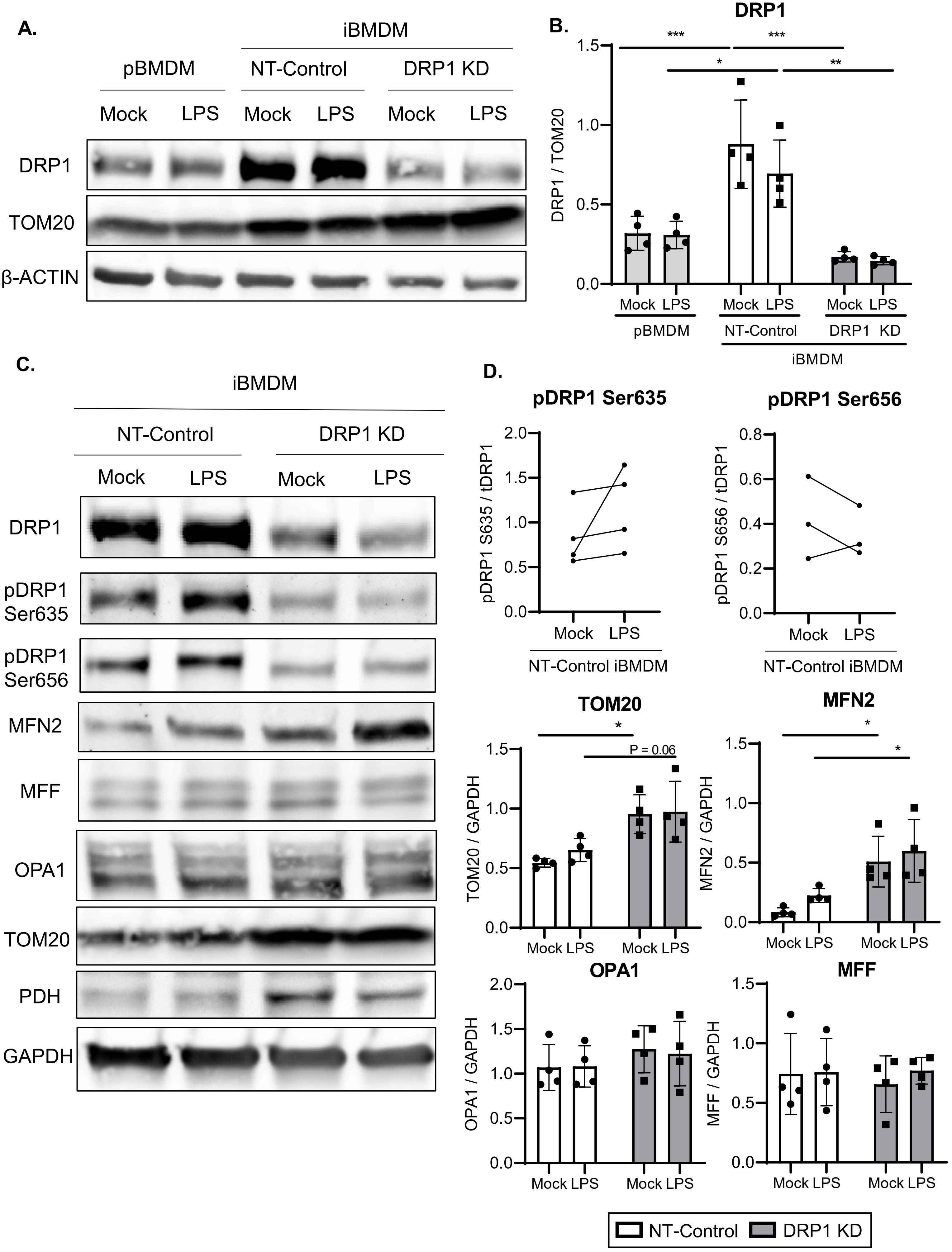

A.
iBMDM
pBMDM
NT-Control
DRP1 KD
Mock
LPS
Mock
LPS
Mock
LPS
DRP1
TOM20
β-ACTIN
B.
DRP1
***
***
*
**
DRP1 / TOM20
1.5
1.0
0.5
0.0
Mock LPS
Mock LPS
Mock LPS
pBMDM
NT-Control
DRP1 KD
iBMDM
C.
iBMDM
NT-Control
DRP1 KD
Mock
LPS
Mock
LPS
DRP1
pDRP1 Ser635
pDRP1 Ser656
MFN2
MFF
OPA1
TOM20
PDH
GAPDH
D.
pDRP1 Ser635
pDRP1 S635 / tDRP1
2.0
1.5
1.0
0.5
0.0
Mock
LPS
NT-Control iBMDM
pDRP1 Ser656
pDRP1 S656 / tDRP1
0.8
0.6
0.4
0.2
0.0
Mock
LPS
NT-Control iBMDM
TOM20
*
P = 0.06
TOM20 / GAPDH
1.5
1.0
0.5
0.0
Mock LPS
Mock LPS
MFN2
*
*
MFN2 / GAPDH
1.5
1.0
0.5
0.0
Mock LPS
Mock LPS
OPA1
OPA1 / GAPDH
2.0
1.5
1.0
0.5
0.0
Mock LPS
Mock LPS
MFF
MFF / GAPDH
1.5
1.0
0.5
0.0
Mock LPS
Mock LPS
NT-Control
DRP1 KD

Fig. S2

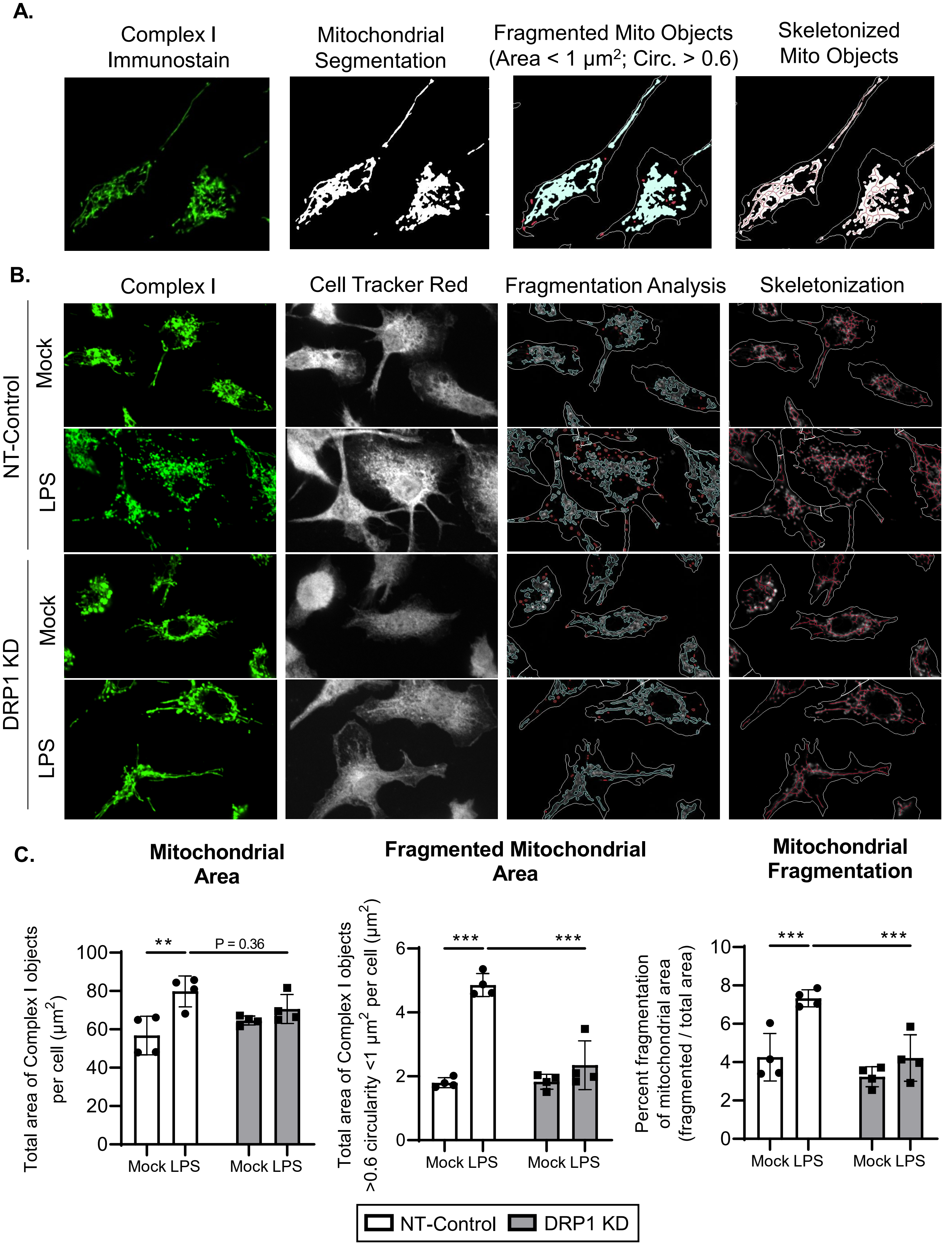
A.
Complex I Immunostain
Mitochondrial Segmentation
Fragmented Mito Objects (Area < 1 μm2; Circ. > 0.6)
Skeletonized Mito Objects
B.
Complex I
Cell Tracker Red
Fragmentation Analysis
Skeletonization
NT-Control
Mock
LPS
DRP1 KD
Mock
LPS
C.
Mitochondrial Area
Total area of Complex I objects per cell (μm2)
**
P = 0.36
100
80
60
40
20
0
Mock LPS
Mock LPS
Fragmented Mitochondrial Area
Total area of Complex I objects >0.6 circularity <1 μm2 per cell (μm2)
***
***
6
4
2
0
Mock LPS
Mock LPS
Mitochondrial Fragmentation
Percent fragmentation of mitochondrial area (fragmented / total area)
***
***
10
8
6
4
2
0
Mock LPS
Mock LPS
NT-Control
DRP1 KD

Fig. S3

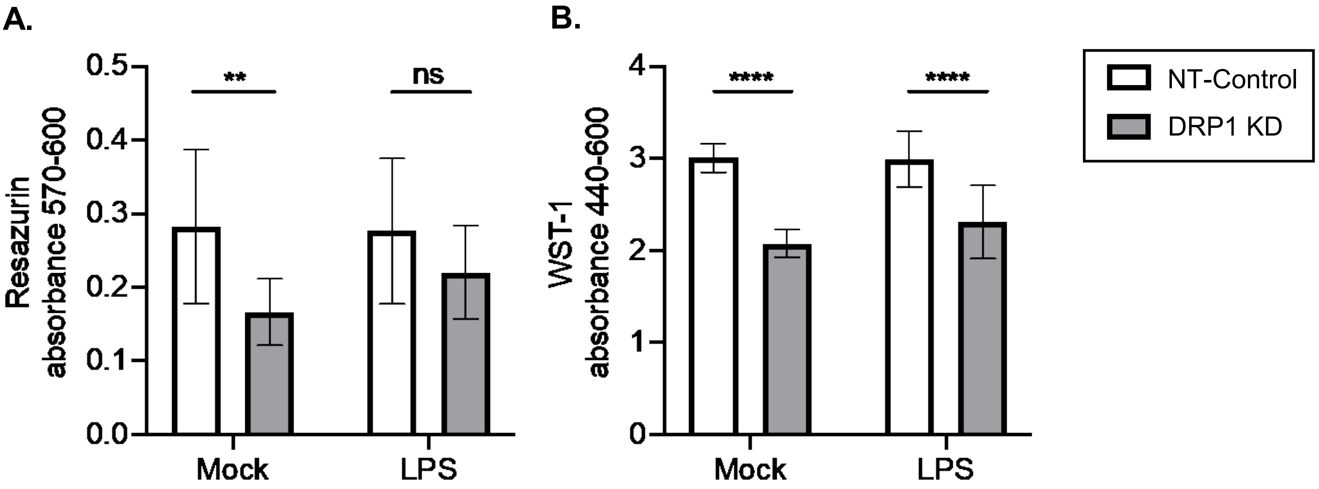
A.
Resazurin absorbance 570-600
0.5
0.4
0.3
0.2
0.1
0.0
**
ns
Mock
LPS
B.
WST-1 absorbance 440-600
4
3
2
1
0
****
****
Mock
LPS
NT-Control
DRP1 KD

Fig. S4

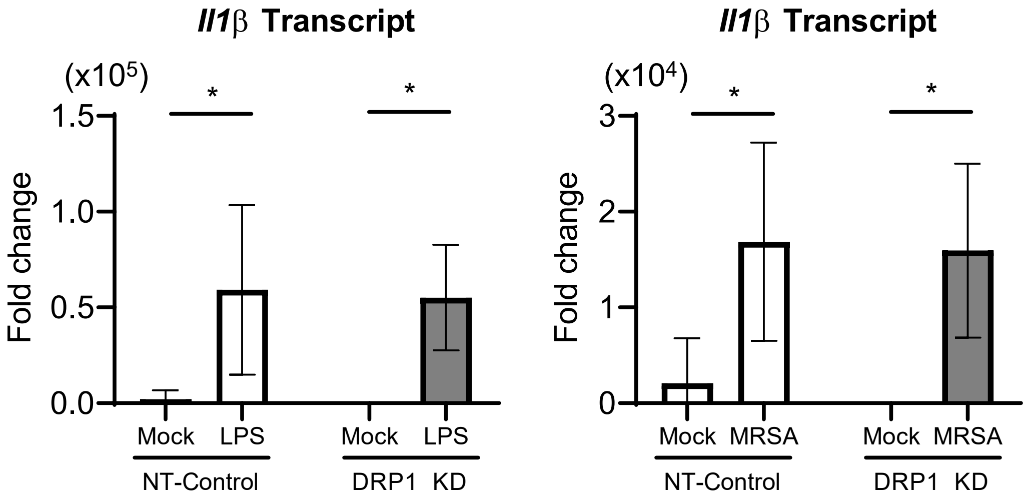

Il1β Transcript
(x10⁵)
Fold change
1.5
1.0
0.5
0.0
*
*
Mock
LPS
Mock
LPS
NT-Control
DRP1 KD
Il1β Transcript
(x10⁴)
Fold change
3
2
1
0
*
*
Mock
MRSA
Mock
MRSA
NT-Control
DRP1 KD

Figure S1. Immunoblot analysis of mitochondrial fission and fusion machinery in macrophages during LPS stimulation. **(A)** Immunoblot comparing DRP1 protein content in primary differentiation BMDM vs. DRP1 KD and NT-Control immortalized BMDM +/- 4h LPS stimulation (200 ng/ml). Lysates from equivalent numbers of cells (7.5×10^4) were loaded to control for cell type-intrinsic differences in protein content. **(B)** DRP1 abundance was quantified relative to mitochondrial content (TOM20) based on immunoblots. **(C)** Analysis of mitochondrial fission and fusion regulators in 4h LPS-stimulated macrophages by immunoblot. The abundance of DRP1 activating phosphorylation at Ser635, DRP1 inhibitory phosphorylation at Ser656, Mitochondrial fission factor (MFF), key mitochondrial fusion proteins, Mitofusion 2 (MFN2) and Optic atrophy 1 (OPA1), and mitochondrial OM marker TOM20 and matrix marker Pyruvate Dehydrogenase (PDH) were assessed. **(D)** Quantification of DRP1 Ser635 and Ser656 phosphorylation relative to total DRP1 (tDRP1) and TOM20, MFN2, OPA1, and MFF relative to GAPDH. Graphs are presented as the mean of n ≥ 3 independent experiments ± SD. Two-way ANOVA with Sidak's multiple comparisons post-test was used to determine statistical significance. **P* < 0.05; ***P* < 0.01; ****P* < 0.001

Figure S2. Quantification of mitochondrial morphology using CellProfiler. **(A)** CellProfiler workflow for mitochondrial fragmentation and skeletonization analysis of Complex I immunofluorescence images. **(B)** Mitochondrial and total cell objects were segmented based on Complex I and Cell Tracker Red CMTPX stains, respectively. Representative processed images of NT-Control and DRP1 KD macrophages showing fragmented mitochondria in red and non-fragmented mitochondrial objects in teal overlaid on Complex I images. Similarly, skeletonized mitochondrial networks (red) are shown overlaid on Complex I images. Cells were left untreated (Mock) or stimulated with LPS (200 ug/ml) for 4h. **(C)** Quantification of mitochondrial fragmentation and length at the cell level. Fragmented mitochondria were classified based on mitochondrial object size and circularity. Mitochondrial objects with area less than 1 μm^2 and a circularity greater than 0.6 were considered fragmented for the purpose of quantification. Cell-level measurements of the number of fragmented mitochondrial objects, percentage of the mitochondrial area classified as fragmented, and the mean length of mitochondrial skeletons are reported as violin plots with the mean designated by a red line. (D) Area of total and fragmented mitochondrial objects per cell are reported as the experimental mean. Similarly, the mean percentage of mitochondrial objects classified as fragmented per cell is reported for each experiment. Graphs are presented as the mean of >100 cells per condition across 4 independent experiments ± SD. Since cell-level data did not follow a normal distribution, *P* values were calculated using direct comparison with the nonparametric Mann-Whitney/Wilcoxon rank sum test. Experimental averages were analyzed with two-way ANOVA with Sidak's post-test for multiple comparisons. ***P* < 0.01; ****P* < 0.001; *****P* < 0.0001

Figure S3. Silencing DRP1 reduces cellular redox. NT-Control and DRP1 KD macrophages were left untreated (Mock) or stimulated with LPS (200 ng/ml) and cellular redox was assessed by using Resazurin **(A)** and WST-1 **(B)** substrates. Graphs indicate mean ± SD of n ≥ 3 independent experiments. Two-way ANOVA with Sidak's multiple comparisons post-test was performed to calculate *P* values. ***P* < 0.01; *****P* < 0.0001

Figure S4. DRP1 knockdown does not affect LPS or MRSA-induced *Il1β* transcription in macrophages. DRP1 KD and NT-Control macrophages were stimulated for 4h +/- LPS (200 ng/ml) or MRSA (MOI 20) and then the levels of *Il1β* transcript were analyzed by RT-qPCR. Data were normalized against NT-Control macrophage untreated (Mock) condition. Graphs indicate mean ± SD of n ≥ 3 independent experiments. Two-way ANOVA with Sidak's multiple comparisons post-test was performed to calculate *P* values. **P* < 0.05
